# A framework for knowledge translation linked to a national clinical guidance initiative

**DOI:** 10.1186/1472-6963-14-S2-P142

**Published:** 2014-07-07

**Authors:** Linda Young, Craig Ramsay, Maria Prior, Gillian Forbes, Anna Templeton, Jan Clarkson

**Affiliations:** 1 TRiaDS, NHS Education for Scotland, Dundee, DD1 4HN, UK; 2 HSRU, University of Aberdeen, Aberdeen, AB24 3FX, UK; 3 DHSRU, University of Dundee, Dundee, DD1 4HN, UK

## 

Clinical guidance is a common strategy to help promote knowledge translation (KT). However, it has been demonstrated that the simple publication of guidance is unlikely to optimise practice. For dentistry in Scotland, the production of national clinical guidance is the responsibility of the Scottish Dental Clinical Effectiveness Programme (SDCEP). To support the translation of SDCEP guidance into practice, TRiaDS (Translation Research in a Dental Setting), a multidisciplinary research collaboration, has developed a standardised KT framework embedded within the guidance development process. The TRiaDS framework is underpinned by the COM-B system [1] and Theoretical Domains Framework [2] to help understand context and the nature of the behaviour(s) to be changed, and to guide identification of behavioural target(s) and potential interventions for change.

For each guidance, a diagnostic analysis begins at the start of guidance development. Key recommendations and associated behaviours are identified and prioritized. Information is gathered about current practice to help quantify any recommendation practice gaps. Questionnaires, focus groups and interviews are used to identify barriers and enablers to change. Where possible, routinely collected data are used to measure compliance with recommendations and inform decisions about whether a KT intervention is required. If an intervention is not required, continued compliance is monitored. If an intervention is required, it is evaluated using an experimental or quasi-experimental design.

The TRiaDS framework has been applied to eight guidance topics. Findings have informed the guidance development process for all. For three topics (decontamination; oral health assessment; drug prescribing), KT interventions have been evaluated in randomised controlled trials embedded within routine service delivery.

The TRiaDS framework enables a timely assessment of the impact of each SDCEP guidance document and a theoretically informed approach to the need for and choice of additional KT interventions. TRiaDS informs dental practitioners, policy-makers and patients on how best to translate guidance recommendations into routine clinical activities. In addition, although based in primary dental care and focused on SDCEP guidance, the generalizability of the TRiaDS framework is being explored within primary care optometry and pharmacy.
